# Risk factors related with high sodium intake among Malaysian adults: findings from the Malaysian Community Salt Survey (MyCoSS) 2017–2018

**DOI:** 10.1186/s41043-021-00233-2

**Published:** 2021-05-31

**Authors:** Nur Shahida Abdul Aziz, Rashidah Ambak, Fatimah Othman, Feng J. He, Muslimah Yusof, Faizah Paiwai, Suhaila Abdul Ghaffar, Muhammad Fadhli Mohd Yusof, Siew Man Cheong, Graham MacGregor, Tahir Aris

**Affiliations:** 1 Institute for Public Health, National Institutes of Health, Ministry of Health Malaysia, Kuala Lumpur, Selangor Malaysia; 2 Wolfson Institute of Preventive Medicine, London School of Medicine & Dentistry, Queen Mary University of London, London, UK; 3 Paediatric Department, Women and Children Hospital, Kuala Lumpur, Malaysia

**Keywords:** High sodium, Associated factors, Adults, Sodium intake, Malaysia, MyCoSS

## Abstract

**Background:**

High sodium intake was an established risk factor for stroke and cardiovascular diseases. The objective of this study was to investigate factors associated with high sodium intake based on 24-h urinary sodium excretion from the MyCoSS study.

**Methods:**

The cross-sectional survey was conducted among adults aged 18 years and above in Malaysia. A multi-stage stratified sampling was used to represent nationally. Twenty-four-hour urine was collected from a total of 900 respondents. Indirect ion-selective electrode (ISE) method was used to measure sodium intake. Descriptive and logistic regression analysis was applied to determine factors associated with high sodium intake based on 24-h urinary sodium excretion.

**Results:**

A total of 798 respondents (76% response rate) completed the 24-h urine collection process. Logistic regression revealed that high sodium intake associated with obese [aOR 2.611 (95% CI 1.519, 4.488)], male [aOR 2.436 (95% CI 1.473, 4.030)], having a waist circumference of > 90cm for adult males [aOR 2.260 ( 95% CI 1.020, 5.009) and >80cm for adult females [aOR 1.210 (95% CI 0.556, 2.631)], being a young adult [aOR 1.977 (95% CI 1.094, 3.574)], and living in urban areas [aOR 1.701 (95% CI 1.094, 2.645)].

**Conclusion:**

Adults who are obese, have a large waist circumference, of male gender, living in urban areas, and belonging to the young adult age group were found to have higher sodium intake than other demographic groups. Hence, reduction of salt consumption among these high-risk groups should be emphasised to reduce the risk of cardiovascular diseases.

## Background

Excessive sodium consumption is a leading cause of high blood pressure, which can lead to greater risk of stroke and cardiovascular disease (CVD) [1, 2]. About 12.8% of all death globally was from high blood pressure [3]. Hence, based on the evidence of the beneficial effects of salt reduction, the World Health Organization (WHO) recommends a daily population intake for sodium of <2.0 g per day (5 g per day of salt) [1].

Therefore, from a preventive medicine perspective, reducing the amount of sodium intake will help in reducing the level of blood pressure. Studies have shown lower sodium consumption was associated with lower systolic blood pressure(SBP) and diastolic blood pressure (DBP), and it could potentially reduce the prevalence of hypertension and other health consequences [4–6]. Moreover, risk reduction in stroke was 4.2% and 3.8% for myocardial infarction (MI) when systolic blood pressure is reduced by 2 mmHg [7]. The Dietary Approach to Stop Hypertension (DASH) study showed that lowering sodium in diet had an even stronger impact on reducing blood pressure and this study contributed much of the scientific basis for the Dietary Guidelines for Americans 2015–2020. The guideline recommended less than a teaspoon of sodium per day [8]. However, there were several factors which lead to high sodium intake such as age, social, culture, education, and income level [9].

The 24-h urinary excretion test is a widely used and valid tool for assessing dietary sodium intake [10]. It is also known as the gold standard method for the assessment of urine excretion. Numerous studies found that most of the sodium consumed in a day (90 to 95%) is excreted through urine in the next 24 h [11, 12]. Cogswell et al. (2018) studied 24-h sodium excretion and blood pressure within and across populations aged 20–59 years old and found that mean sodium excretion was higher in men (4205 mg) than in women (3039 mg) [5].

In the Malaysian context, a few studies have been carried out on sodium consumption for specific groups and found that sodium consumption is high among Malaysians and far exceeds the recommended values [13]. A study among Ministry of Health staff showed that sodium consumption exceeded 2000 mg/day [14]. We aimed to investigate factors that are associated with high sodium intake based on 24-h urinary sodium excretion among a large, nationally representative sample of the adult population in Malaysia.

## Methods

### Study design

This is a cross-sectional household survey that applied a stratified cluster sampling method. to ensure national representativeness in Malaysia which included adults aged more than 18 years old. Data collection was conducted between October 2017 and March 2018. Details of MyCOSS’s methodology are described in an article in the present journal supplement (Introduction to the Malaysian Community Salt Survey (MyCoSS): a population-based survey on salt intake to support salt reduction strategies in Malaysia). Respondents were given a patient information sheet (PIS) in Malay or English language to read prior to obtaining consent. Any queries about the study would be addressed by the study team. Written consent was obtained from all eligible respondents. Respondents who were found to have higher blood pressure or other abnormal results not previously diagnosed or investigated were referred to the nearest government clinic or hospital for further management. The respondents were informed the results of the physical examination, such as blood pressure, height, and weight, during data collection.

Sample size calculation used a formula for estimating population prevalence. The sample size calculation for determination of sodium intake referred to finding from salt study among health workers in Malaysia (My Salt 2015) [15]. Mean sodium of 142 mmol, relative standard error (RSE) of 0.05, and design effect 1.5, including 50% nonresponse, 816. Samples with no missing urine and more than 500 ml were included in analysis. Respondents who were pregnant, suffering from chronic kidney failure, or undergoing dialysis were excluded from this study.

### Data collection

The respondents were given both oral and printed instructions on how to accurately collect the urine. Twenty-four-hour urine collection was used as it was the gold standard for estimating sodium intake. Respondents were required to collect a single urine sample for 24 h, in a 5-l urine container. Plastic cups were given to respondents to assist in collecting urine. They were instructed to discard the first urine void in the morning and collect all subsequent urine until the first urine void in the following morning. The last collection was the first urination on the second day of collection. They recorded the beginning and end of 24-h urine collection, and were asked to notify any missed collection to data collectors. Sodium was measured using the indirect ion-selective electrode (ISE) method [10]. Sodium intake (mg) in a day was calculated by multiplying 24-h urinary sodium excretion (mmol) with a factor of 23. Urine results were given to the respondents at the end of the study along with recommendations on reducing salt intake. Sodium intake was then compared to the Malaysian and WHO dietary sodium recommended intake of less than 2000 mg/day.

### Statistical analysis

Population estimates were made by weighting the age and sex specific estimates obtained from the survey using sample-to-population weights calculated using census data. Descriptive statistics of complex sample data was computed and mean sodium intake was compared within group of socio-demography and anthropometry characteristic. Significant *p* values were at less than 0.05. Complex sample logistic regression analysis was used to determine the association between risk factors related with high sodium intake (defined as 24-h sodium excretion exceeding 2000mg/day). Crude odds ratios (cOR) were used to describe the strength of association between factors and high sodium intake. The adjusted OR (aOR), with *p* value less than 0.05, was considered significant in the full logistic regression model. All statistical analyses were conducted using SPSS version 21.

## Results

A total of 798 urine samples were collected from respondents. About 79% of the Malaysian population consumed sodium excessively in their diet. Table 1 shows the basic socio-demographic characteristics. Mean sodium intake was higher among respondents in urban areas (3252.3 mg/day) compared in rural areas. Based on gender, men (3519 mg/day) showed significantly higher sodium intake compared to women. Among age groups, younger adults 25–34 years old (3556 mg/day) showed significantly higher sodium intake compared to other age groups. Besides that, never married/single (3389.7mg/day) had higher mean sodium intake as compared with other marital status while self-employed (3467.2 mg/day) had higher mean sodium intake compared to other types of occupation. Meanwhile for nutritional status by BMI cut off points, obese respondents (3643.2 mg/l) had significantly higher mean sodium intake compared to other categories.

**Table 1:** Sodium intake (mg/day) by socio-demographic and anthropometric characteristics of respondents (*n*=798)

	Unweighted count	%	Sodium intake		
			Mean	95% CI	
				Lower	Upper
**Malaysia**	798	100.0	3166.7	2987.33	3346.10
**Strata**					
Urban	319	75.8	3252.3	3022.82	3481.82
Rural	479	24.2	2895.9	2749.57	3042.16
**Gender**					
Men	340	51.7	3519.5	3210.00	3828.92
Women	458	48.3	2789.2	2648.86	2929.52
**Age groups (years)**					
18–39	235	29.4	3176.7	2983.54	3370.05
40–59	347	43.5	2985.1	2840.10	3130.11
60+	216	27.1	2823.2	2641.76	3004.56
**Marital status**					
Never married	93	11.5	3389.7	3021.7 3	3757.7 4
Married	594	77.7	3057.2	2946.24	3168.2 0
Separated/widower	110	2.5	2340.9	2154.02	2527.78
**Education level**					
None	64	5.1	2745.2	2125.37	3346.94
Primary	167	18.2	3051.4	2666.83	3435.97
Secondary	383	51.1	3234.6	2990.52	3478.77
Tertiary	184	25.6	3207.0	2956.47	3457.44
**Occupation**					
Public	117	14.0	3324.7	2871.13	3778.40
Private	126	17.2	3337.0	2989.19	3684.88
Self-employed	179	23.0	3467.2	3036.29	3898.10
Housewives	214	23.5	2903.6	2720.87	3086.43
Unemployed	113	13.7	2887.2	2541.58	3243.01
Student	15	2.0	2919.6	2182.25	3653.12
Others	34	6.6	2919.4	2425.44	3409.36
**Body mass index (kg/m**^**2**^**)**					
Underweight	35	4.5	2028.5	1697.20	2359.88
Normal	285	35.2	2878.0	2672.62	3083.47
Overweight	287	37.5	3283.2	3005.98	3560.45
Obese	191	22.8	3643.2	3216.13	4070.86
**Waist circumference (cm)**					
Men <90	166	34.7	3033.3	2767.10	3299.55
Men >90	174	65.3	3901.0	3450.85	4351.17
Women <80	180	39.3	2462.1	2192.16	2732.22
Women ≥80	618	60.7	2887.2	2887.21	3046.36

Logistic regression analysis showed high sodium intake was significantly associated with strata, gender, age group, waist circumference, and body mass index (Table 2). Specifically, being obese [aOR 2.611 (95%CI 1.519, 4.488)] and having a waist circumference >90cm for males [aOR 2.260 ( 95%CI 1.020, 5.009)] and >80cm for females [aOR 1.210 (95%CI 0.556, 2.631)] were significantly more likely to have high sodium intake. Besides, being male [aOR 2.436 (95% CI 1.473, 4.030)], being young adults [aOR 1.977 (95%CI 1.094, 3.574)], and living in urban areas [aOR 1.701 (95% CI 1.094, 2.645)] were also more likely to have high sodium intake.

**Table 2:** Factors associated with high sodium intake among Malaysian adults (*n*=798)

Study characteristic	Unadjusted			***p*** value	Adjusted			***p*** value
	Crude OR	95%CI			Adjusted OR	95%CI		
		Lower	Upper			Lower	Upper	
**Strata**								
Urban	1.730	1.198	2.499	0.039	1.701	1.094	2.645	0.017*
Rural	1	-	-		1	-	-	
**Gender**								
Men	2.013	1.183	3.426	<0.001	2.436	1.473	4.030	<0.001*
Women	1	-	-		1	-	-	
**Age groups (years)**								
18–39	1.588	0.889	2.835	0.027	1.977	1.094	3.574	0.010*
40–59	1.707	1.006	2.897	0.045	1.871	1.136	3.081	0.043*
60+	1	-	-		1	-	-	
**Body mass index (kg/m**^**2**^**)**								
Underweight	0.347	0.138	0.874	0.078	0.2281	0.071	0.726	0.107
Normal	1	-	-		1	-	-	
Overweight	22.106	1.344	3.300	<0.001	1.890	1.111	3.214	0.007*
Obese	2.659	1.619	4.368	<0.001	2.611	1.519	4.488	<0.001*
**Waist circumference (cm)**								
Men <90	1	-	-		1	-	-	
Men >90	2.311	0.998	5.353	0.018	2.260	1.020	5.009	<0.001*
Women <80	1	-	-		1	-	-	
Women ≥80	1.597	0.860	2.967	0.045	1.210	0.556	2.631	0.049*

*Significance at *p* value < 0.05

## Discussion

Mean sodium intake in this study was 3167 mg per day (3167 mg/day = 138 mmol/day = 7.9 g salt = 1.6 tsp.). Besides, high sodium intake was also seen in urban areas, among men and obese respondents. This finding showed that sodium intake among the Malaysian population was higher than the WHO’s recommendation of less than 2000 mg/day of sodium [1]. Similarly other countries such as the USA, the UK, Singapore, and Korea reported that their result of sodium intake based on 24-h urine sodium excretion was higher than the WHO’s recommendation [16, 17].

Several studies have demonstrated evidence that high sodium intake was associated with an increased risk of obesity [12]. This is similar to findings in the present study where respondents with high body mass index (BMI) were more likely to have high sodium intake. This might be due to high consumption of high calorie foods, seasoning, sauces, or other junk foods. Most processed food products are known to have high sodium content as a preservative substance. Some studies have also revealed that consumption of salt enhanced foods has a direct association with obesity [18, 19]. Eating salty food is also associated with an increase in sugar intake as the high sodium intake will induce feeling of thirst leading to increased fluid intake, especially sugar-sweetened beverages [20, 21].

The present study also shows that high sodium intake and a large waist circumference are positively associated [aOR=2.260 (male), 1.210 (female)], comparable to a similar study conducted in Brazil [22]. Respondents with high sodium intake may tend to have greater energy intake and unhealthy food intake which were associated with increased risk for being overweight and abdominal obesity. In addition, high sodium intake also has a strong association with increased BMI [18, 23].

In this study, salt intake was significantly higher among males (3519 mg/day) compared to females (2790 mg/day), which was similar with other studies [4, 12] that revealed males tend to have higher sodium intake [15]. This might be due to the differences in physical structure and calorie intake requirements between both sexes. This finding was also supported by another study that reported males consumed high sodium foods 10% more than females [24]. It may be possible that females are generally more concerned about their physical appearance and health, which compels them to make better and healthier food choices which tend to be less calorie-dense and low in sodium.

Findings from the present study also showed that sodium intake is influenced by age. Younger and middle-aged adults between 18 and 40 years were found to have higher sodium intake than those aged 60 years and above. Similar findings from another study reported that adults aged 30–40 years old had a significantly higher sodium intake compared to other age groups [25–28]. It is also known that younger adults have a higher calorie intake and they mostly eat a wider variety of meals and snacks compared with the elderly [26, 27]. The elderly may have reason to control their sodium because of illness or they may just be more health-conscious [29–31]. This finding may guide intervention efforts in salt reduction to focus upon at-risk populations in terms of highest sodium intake.

This study also revealed that respondents living in urban areas were more likely to have high sodium intake. Similar findings of such differences between urban and rural regions were also reported in Ethiopia and India [27]. The ubiquity of and easy accessibility to fast food restaurants, as well as busier lifestyles that allow less time for cooking healthy low-salt meals at home, were among factors contributing to higher sodium intake [32–34].

To our knowledge, this is the first large-scale survey on sodium intake done in Malaysia in a community setting. However, we were not able to evaluate causality due to the cross-sectional design. Another limitation is that data on drug histories or other dietary components that might affect urinary sodium excretion were not available. Collection of 24-h urine is labour intensive, with low response rates and sample rejection due to missing urine creating potential for bias.

## Conclusions

In summary, the identification of certain socio-demographic factors such as age, sex, locality, BMI, and waist circumference that are strongly associated with high sodium intake provides important information to be taken into consideration in developing interventions for reducing sodium intake among the Malaysian population.

## Data Availability

The datasets used and/or analysed during the current study are available from the corresponding author on reasonable requests.

## Abbreviations

BMI: Body mass index
ISE: Ion selective electrode
SBP: Systolic blood pressure
DBP: Diastolic blood pressure
aOR: Adjusted odds ratio
OR: Odds ratio
WHO: World Health Organization
DASH: Dietary Approaches to Stop Hypertension
